# SCD1 Confers Temozolomide Resistance to Human Glioma Cells via the Akt/GSK3β/β-Catenin Signaling Axis

**DOI:** 10.3389/fphar.2017.00960

**Published:** 2018-01-04

**Authors:** Shuang Dai, Yuanliang Yan, Zhijie Xu, Shuangshuang Zeng, Long Qian, Lei Huo, Xuejun Li, Lunquan Sun, Zhicheng Gong

**Affiliations:** ^1^Department of Pharmacy, Xiangya Hospital, Central South University, Changsha, China; ^2^School of Pharmaceutical Sciences, Central South University, Changsha, China; ^3^National Clinical Research Center for Geriatric Disorders, Xiangya Hospital, Central South University, Changsha, China; ^4^Department of Pathology, Xiangya Hospital, Central South University, Changsha, China; ^5^Department of Neurosurgery, Xiangya Hospital, Central South University, Changsha, China; ^6^Center for Molecular Medicine, Key Laboratory for Molecular Radiation Oncology of Hunan Province, Xiangya Hospital, Central South University, Changsha, China

**Keywords:** glioblastomas, temozolomide, resistance, SCD1, Akt signaling

## Abstract

Resistance to temozolomide (TMZ), the standard chemotherapy agent for glioblastoma (GBM), poses a major clinical challenge to GBM prognosis. Understanding the mechanisms of TMZ resistance can help to identify novel drug targets and more effective therapies. Recent studies suggest that bioenergetic alterations of cancer cells play important roles in drug resistance. In our study, the altered metabolism of cancer cells was observed using a metabolic PCR array. We found that stearoyl-coenzyme A desaturase 1 (SCD1), a key rate-limiting enzyme for synthesis of monounsaturated fatty acids, was significantly upregulated in TMZ-resistant GBM cells compared to their parental counterparts. Overexpression of SCD1 promoted resistance to TMZ in parental GBM cells, whereas SCD1 downregulation by siRNA could re-sensitize TMZ-resistant cells *in vitro*. Combinational treatment of TMZ and an SCD1-specific inhibitor showed a combined inhibitory effect on TMZ-resistant glioma cells. We also observed that overexpression of SCD1 promoted Akt/GSK3β/β-catenin signaling, while silencing of SCD1 inhibited the signaling. The combination of an Akt activator with exogenous SCD1 or the combined inhibition of Akt and enforced expression of SCD1 resulted in the most significant changes of Akt signaling. Functionally, significantly lower viability and mobility rates were observed in TMZ-resistant cells when treated with Akt inhibitors and an SCD1 inhibitor simultaneously compared to when treated individually. In conclusion, our study identified SCD1 along with its functional pathway as a novel target in the development of TMZ resistance. SCD1 inhibition used alone or in combination with Akt inhibition could effectively overcome TMZ resistance in gliomas.

## Introduction

Glioblastoma (GBM), the most common and lethal form of primary intrinsic brain tumors in adults, has the highest mortality rate among all malignant brain and CNS tumors. Maximal surgical resection followed by radiotherapy plus daily temozolomide (TMZ) has been widely recognized as the standard treatment regimen for patients with newly diagnosed glioblastoma since 2005. Despite remarkable advances in GBM treatment, the clinical prognosis remains poor with a median overall survival of 15–17 months (Wang et al., 2016; Yan et al., 2017). TMZ, a second-generation imidazotetrazine prodrug, has high bioavailability and tolerability and transports well across the blood-brain-barrier (BBB), providing modest antitumor activity against GBM (Shi et al., 2014; Stupp et al., 2015; Baumert et al., 2016). However, recent studies have indicated that a majority of patients with GBM gradually develop resistance to TMZ during treatment.

Various mechanisms of TMZ resistance has been previously discussed, involving DNA repair systems [e.g., *O*-6-methylguanine-DNA methyltransferase (MGMT)], abnormally mutated genes [e.g., epidermal growth factor receptor (EGFR), p53], and activated protein kinase B (PKB/Akt) signaling pathway (Yan et al., 2016; Nie et al., 2017). To date, metabolic reprogramming has been demonstrated to be one of the important hallmarks of cancer biology. Meanwhile, emerging evidence suggests that altered metabolism in cancer cells is fundamentally involved in the development of drug resistance (Pavlova and Thompson, 2016). Zhao et al. (2011) reported that lactate dehydrogenase-A (LDH-A) is significantly upregulated and activated in taxol-resistant breast cancer cells. LDH-A knockdown by siRNA could re-sensitize taxol-resistant breast cancer cells to taxol (Zhou et al., 2010). This same group also found that the increased glycolysis mediated by HSF1 and LDH-A overexpression contributes to trastuzumab resistance, and the combination of trasuzumab and glycolysis inhibition synergistically inhibits the growth of both trasuzumab-sensitive and -resistant breast cancer cells *in vitro* and *in vivo* (Zhao et al., 2011). ATP citrate lyase, the first and rate-limiting step for *de novo* lipogenesis, was also found to mediate SN38 resistance in colorectal cancer cells (Zhou et al., 2013). These findings suggest that targeting key metabolic enzymes could provide promising strategies for improving treatment efficacy.

Stearoyl-coenzyme A desaturase 1 (SCD1) is a key rate-limiting enzyme responsible for the synthesis of monounsaturated fatty acids (MUFAs). Accumulating evidence has shown that SCD1 plays critical roles in the progression, survival, differentiation, and transformation of human cancers (Igal, 2016). With predominantly tumor-promoting properties, SCD1 has been noted to be upregulated in multiple cancers, including lung adenocarcininoma (Huang et al., 2016), hepatocellular carcinoma (Huang et al., 2015), and clear cell renal carcinoma (von Roemeling et al., 2013). The critical role of SCD1 in regulating cancer cell phenotype was clearly demonstrated by loss-of-function studies in neoplastic cells. The ablation of SCD1 expression using siRNA or specific inhibitors dramatically reduces cell proliferation and invasion, and impairs tumor formation (Sanchez-Martinez et al., 2015). However, the contribution of SCD1 to drug resistance of cancer cells remains to be elucidated.

In this study, we performed a PCR array to evaluate metabolism reprogramming in the development of TMZ resistance in gliomas. Our results showed that SCD1 is the most notably upregulated gene in established TMZ-resistant GBM cells. Furthermore, we investigated the association between SCD1 activity and TMZ chemosensitivity, as well as SCD1’s functional and mechanistic roles in mediating TMZ resistance. Our findings revealed that SCD1 plays a pivotal role in TMZ-resistant GBM, and that targeting SCD1 could re-sensitize TMZ-resistant GBM cells through the Akt/GSK3β/β-catenin signaling axis.

## Materials and Methods

### Cells and Reagents

The TMZ-resistant glioma cell lines, T98G-R and U87-R, were derived from the parental cell lines (T98G and U87) by treatment with gradually increasing concentrations of TMZ. Human malignant glioma cell lines T98G, U87, U251, U343, MGR2, and Hs683 were cultured in high-glucose DMEM medium (Glibco, United States), supplemented with 10% (v/v) fetal bovine serum (HyClone, United States), 1% penicillin and streptomycin. All cell lines were grown in a humidified incubator at 37°C with 5% CO_2_.

Temozolomide and epidermal growth factor (EGF) were purchased from Sigma–Aldrich Corporation Chemicals. A939572 and LY294002 were purchased from MedChem Express. MK2206 was purchased from Selleck Chemicals. All reagents above were dissolved in dimethylsulfoxide (DMSO) (Sigma). For the Akt activation, cells were starved by serum-free medium incubation for 24 h, and then treated with 30 ng/mL EGF for 30 min. The exposed concentrations of TMZ, MK2206, and LY294002 were 200, 5, and 20 μM, respectively.

### Cell Transfection

An SCD1 overexpression plasmid was synthesized by cloning human SCD1 cDNA into plasmid pcDNA3.1 (pcDNA-SCD1), and the empty plasmid pcDNA3.1 served as the vector control. Small interfering RNA (siRNA) targeting SCD1 (si-SCD1, 5′-GCACAUCAACUUCACCACATT-3′) was purchased from Genepharma (Suzhou, China), with a scrambled siRNA (si-Ctrl, 5′-UUCUCCGAACGUGUCACG UTT-3′) used as the negative control. Transfection was performed using Lipofectamine 3000 reagent (Invitrogen, Carlsbad, CA, United States) according to the manufacturer’s protocol.

### RNA Extraction and Quantitative PCR (qPCR)

Total RNA was extracted from glioma cell lines using TRIzol reagent (Invitrogen, Carlsbad, CA, United States) according to the manufacturer’s protocol, and reverse transcribed to cDNA using the PrimeScript^TM^ RT reagent kit (Takara, Dalian, China). The qPCR assay was carried out using iTaq^TM^ Universal SYBR green Supermix (Bio-Rad, United States), with β-actin as the internal control. The forward and reverse primer sequences were used as follows: β-actin: 5′-CATGTACGTTGCTATCCAGGC-3′ and 5′-CTCCTTAATGTCACGCACGAT-3′; MGMT: 5′-ACCGTTTGCGACTTGGTAC TT-3′ and 5′-GGAGCTTTATTTCGTGCAGACC-3′; SCD: 5′-TCTAGCTCCTATACCACCACCA-3′ and 5′-TCGTCTCCAACTTATCTCCTCC-3′. Relative expression levels were determined with the 2^-ΔΔ*C*_T_^ method. All reactions were run three or more times.

### Western Blot Analysis

The antibodies for western blotting were provided as follows: α-Tubulin (sc-69969, Santa Cruz), SCD1 (ab193332, Abcam), β-catenin (Cell Signaling Technology), GSK3β (sc-8257, Santa Cruz), p-GSK3β (Ser9) (9323s, Cell Signaling Technology), Akt (9272s, Cell Signaling Technology), p-Akt (4060, Cell Signaling Technology), γ-H2AX (7631, Cell Signaling Technology), E-cadherin (3195s, Cell Signaling Technology), and Vimentin (5741p, Cell Signaling Technology). Forty micrograms of each total protein sample was separated by 6–10% SDS-PAGE, transferred onto the surface of polyvinylidene fluoride membrane and immunoblotted with the indicated primary antibodies. Using the enhanced chemiluminescence reagent (Thermo Scientific Pierce ECL, United States), visualization of the protein bands was conducted in the ChemiDoc XRS system (Bio-Rad, Berkeley, CA, United States).

### MTS Assay

Cell viability and cell survival were analyzed by a MTS assay. 24 h after transfection, cells were seeded in 96-well plates at a density of 3 × 10^3^/well, and then treated with various concentrations of TMZ (0, 3.2, 16, 80, 400, 2000 μM) for another 72 h. After a 2 h incubation with 20 μl MTS solution (Dojindo, Kumamato, Japan), the degree of cell survival was detected at 490 nm using a microplate reader (BioTek, Winooski, VT, United States). And the survival rate was calculated by the following formula: Survival rate (%) = (mean OD_treated groups_ – mean OD_blank controls_)/(OD_control groups_ – OD_blank controls_) × 100. The survival of untreated cells was set at 100% and was used to calculate the half-maximal inhibitory concentration (IC_50_). Cell proliferation was determined by treating glioma cells (2000/well) with 200 μM TMZ for 5 days in 96-well plates. The experiments were conducted for at least three replicates and each experiment was performed at least twice.

### Flow Cytometry

First, 1 × 10^6^ cells were seeded in 60 mm dishes overnight, and the cells were cultured with serum-free culture for 24 h. The culture was replaced with 10% FBS high glucose DMEM complete medium containing 200 μM TMZ for 24 h. GBM cells were digested using trypsin and collected, washed twice with 1× PBS buffer and mixed with 1 ml 70% ethanol. Then cells were incubated overnight at 4°C. Collected cells were stained with 1 μL 100 mg/ml RNase and 4 μL propidium iodide (PI) in 200 μL 1× PBS buffer. Samples were incubated at room temperature in the dark for 30 min, and then immediately analyzed by Bio-Rad flow cytometry.

### Wound Healing Assay

The cells were seeded at 1.0 × 10^6^ cells/well in 6-well plates and cultured to at least 95% confluence. Cells in a monolayer were scraped with a plastic 100 μL pipette tip, washed three times in PBS and incubated with FBS-free DMEM medium. The scratched areas were photographed by phase-contrast microscopy after incubation for 0, 12, or 24 h. The relative wound closure was calculated using ImageJ software.

### Fluorescence Confocal Microscopy

The parental and TMZ-resistant glioma cells were grown on Millicell EZ Slide (Millipore) and transfected with SCD1 siRNA or overexpression plasmid for 48 h. After that, cells were fixed with pre-cooled methanol (-20°C). These coverslips were incubated with the γ-H2AX antibody, followed by fluorochrome-conjugated secondary antibody. Meanwhile, the fragmentation of cell nuclei were observed by 4′,6-diamidino-2-phenylindole (DAPI, Thermofisher, D1306). For fluorescence analysis, cell samples were visualized on a laser scanning confocal microscopy with appropriate emission filters (Olympus).

### Statistical Analysis

All experiments were performed in at least triplicate with mean ± SD subjected to Student’s *t*-test for pairwise comparison or ANOVA for multivariate analysis. Data analysis was performed using Graphpad Prism 5 software and the Spearman rank correlation was analyzed by OriginLab. ^∗^*p* < 0.05, ^∗∗^*p* < 0.01, and ^∗∗∗^*p* < 0.001 were considered to be significant for all of the tests.

## Results

### Establishment and Characterization of TMZ-Resistant GBM Cell Lines

**FIGURE 1:**
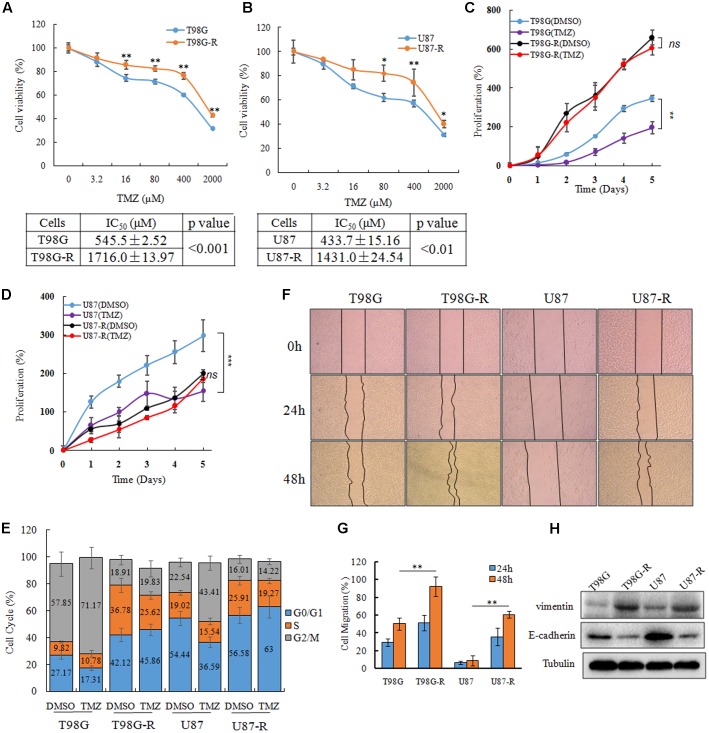
Establishment and characterization of TMZ-resistant GBM cell lines. **(A,B)** Cell viability analysis was performed to evaluate cytotoxicity of TMZ to T98G, T98G-R, U87, and U87-R cells under treatment with the indicated concentrations of TMZ for 72 h. **(C,D)** *In vitro* cell proliferation curves in response to TMZ treatment. **(E)** GBM cells were treated with or without TMZ for 48 h and cell cycle was examined by flow cytometry. **(F,G)** GBM cells subjected to wound healing assays. Cell migration rate was analyzed by ImageJ. **(H)** Expression of E-cadherin and Vimentin in GBM cells was examined by western blot. Above experiments were repeated three independent times with similar results. Each data point represents the mean ± SD. ^∗^*p* < 0.05, ^∗∗^*p* < 0.01, and ^∗∗∗^*p* < 0.001.

To establish TMZ-resistant cell lines, T98G and U87 cells were exposed to increasing concentrations of TMZ (6.25, 12.5, 25, 50, 100 μM), and each concentration was maintained for at least 15 days. The chemoresistant GBM cell lines, T98G-R and U87-R, were generated after chronic exposure to TMZ for 6 months and were maintained in a clinical dose (100 μM) of TMZ. TMZ resistance was confirmed by an MTS assay, flow cytometry, and a wound healing assay. The IC_50_ values of TMZ-resistant GBM cells (T98G-R: 1716.0 ± 13.97 μM, U87-R: 1431.0 ± 24.54 μM) were increased by nearly threefold compared to those of parental GBM cells (T98G: 545.5 ± 2.52 μM, U87: 433.7 ± 15.16 μM) (**Figures 1A,B**). To compare the survival capacity of parental and TMZ-resistant GBM cells, the cells were treated with 200 μM of TMZ for 5 days. We observed that T98G and U87 showed significant tumor proliferation inhibition, while T98G-R and U87-R cells showed no obvious proliferative changes under the TMZ medium exposure (**Figures 1C,D**). Using cell cycle analysis, we observed that TMZ considerably increased the percentage of T98G and U87 cells in G2/M phases and decreased the fraction of cells in G0/G1 phases compared to DMSO-treated cells. However, there was little change on the cell cycle distributions of T98G-R and U87-R cells after TMZ treatment (**Figure 1E**). In addition, it has been previously shown that the chemoresistant cancer cells exhibit a mesenchymal phenotype with increased migration and invasion capacity compared with the parental cells (Tso et al., 2015). Therefore, we confirmed the migration capacity of T98G-R and U87-R cells, and similarly found significant reduction of the scratch marks compared to that of T98G and U87 cells (**Figures 1F,G**). Meanwhile, the protein expression of E-cadherin, a migration inhibitor, and Vimentin, a pro-migration factor (Liu et al., 2017), was also examined by western blot to estimate the degree of epithelial-to-mesenchymal transition (EMT). As expected, we found that T98G-R and U87-R displayed a lower level of E-cadherin and much higher level of Vimentin compared to their parental T98G and U87 cells (**Figure 1H**). These results demonstrated that we successfully established TMZ-resistant cells that had acquired the resistant phenotype.

### SCD1 Is Significantly Overexpressed in TMZ-Resistant GBM Cell Lines

**FIGURE 2:**
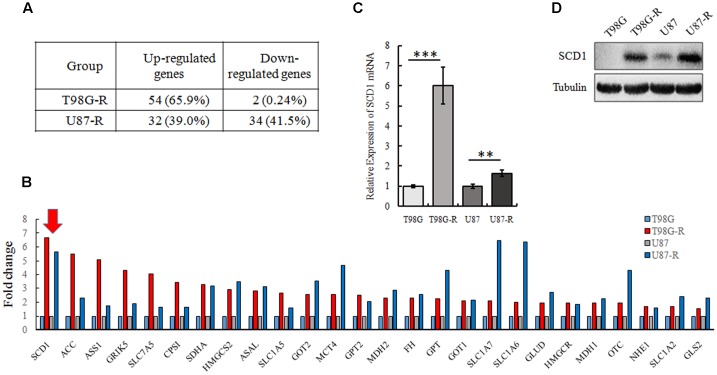
SCD1 is significantly overexpressed in TMZ-resistant GBM cell lines. **(A)** The changes of metabolic genes expression in GBM cells were examined by PCR array. **(B)** Upregulated genes encoding metabolic enzyme in T98G-R and U87-R cells. **(C,D)** Expression of SCD1 was analyzed by qPCR and western blot. The quantitative results shown of three independent experiments are means ± SD. ^∗∗^*p* < 0.01, ^∗∗∗^*p* < 0.001.

To illustrate whether metabolic changes are involved in the development of TMZ resistance in GBM cells, 82 metabolic enzymes were selected to conduct a metabolism-focused PCR array in parental and TMZ-resistant GBM cells. The detailed primer information used for the PCR array is listed in **Supplementary Table S1**. Our metabolic PCR array showed that the expression of many metabolic enzymes involved in glycolysis, glutaminolysis, and lipogenesis differs between parental and TMZ-resistant GBM cells. We identified 56 genes (54 upregulated and 2 downregulated) in T98G-R cells and 66 genes (32 upregulated and 34 downregulated) in U87-R cells that were differentially expressed (fold change > 1.5, *p* < 0.05) (**Figure 2A**). Among these differentially expressed genes, 26 genes exhibited increased expression in both T98G-R and U87-R cells (**Figure 2B**). We observed that the largest change was the upregulation of SCD1, a member of the fatty acid desaturase family, in TMZ-resistant GBM cells. We then examined the expression of SCD1 in T98G/T98G-R and U87/U87-R cells by qPCR and western blot. The mRNA and protein levels of SCD1 were found to be significantly upregulated in the T98G-R and U87-R cell lines compared with their respective parental cell lines (**Figures 2C,D**). These results verify the involvement of cellular metabolic alteration in TMZ resistance and indicate a possible role of SCD1 in the regulation of TMZ resistance.

### SCD1 Modulates TMZ Resistance in GBM Cells

**FIGURE 3:**
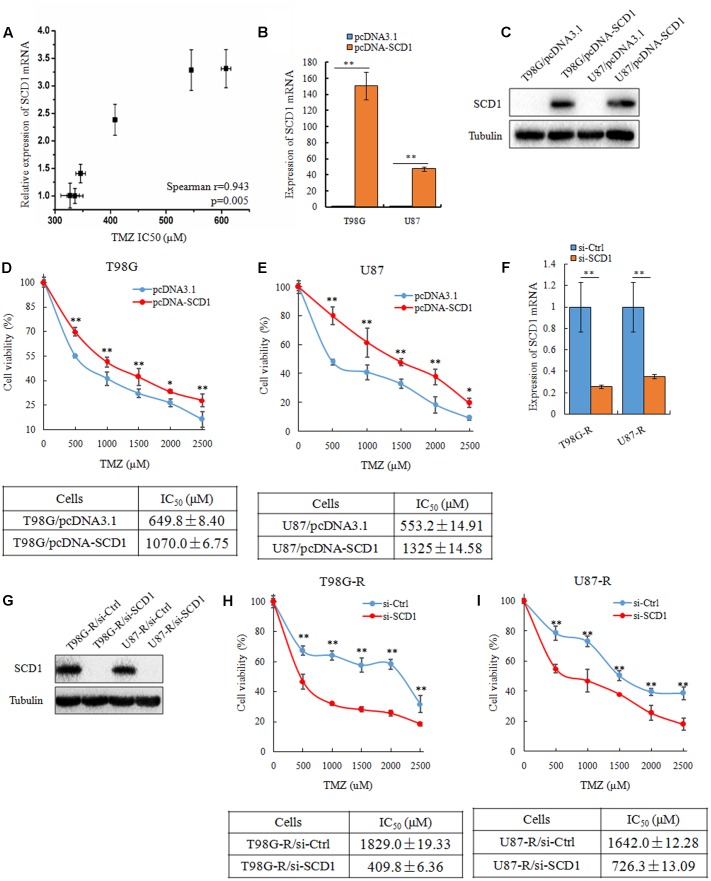
SCD1 modulates TMZ resistance in GBM cells *in vitro*. **(A)** The correlation between SCD1 mRNA expression and IC_50_ values in six glioma cells was quantified by Spearman’s rank correlation. **(B,C)** qPCR and western blot confirmed increased SCD1 expression in T98G and U87 cells 48 h after transfection with SCD1 cDNA clone, versus transfection with the blank plasmid pcDNA3.1. **(D,E)** Cell viability assay was performed in T98G and U87 cells after overexpression of SCD1. **(F,G)** qPCR and western blot show si-SCD1 knockdown after 48 h transfection in T98G-R and U87-R cells. A non-specific siRNA was used in parallel.**(H,I)** T98G-R and U87-R cells transfected with si-SCD1 or si-control subjected to MTS assays. ^∗^*p* < 0.05, ^∗∗^*p* < 0.01. Experiments were performed three times with similar results.

To investigate whether SCD1 expression is associated with chemosensitivity of glioma cells, we examined the mRNA expression of SCD1 and TMZ sensitivity (IC_50_) in six glioma cell lines (U343, Hs683, U251, U87, T87G, MGR2). The correlation between the IC_50_ values and the relative mRNA expression of SCD1 was analyzed using Spearman rank correlation. We observed that the IC50 values of TMZ correlate with the expression level of SCD1 in these glioma cells (Spearman *r* = 0.943, *p* = 0.005) (**Figure 3A**). These data indicate that SCD1 expression could be a predictive marker of TMZ chemosensitivity in glioma cells.

Next, we asked how the expression level of SCD1 affects TMZ chemosensitivity. To address this question, SCD1 was overexpressed in two parental cell lines, T98G and U87 (**Figures 3B,C**). Ectopic expression of SCD1 rendered glioma cells more resistant to TMZ. There was an ∼1.6-fold increase in the IC_50_ of T98G/pcDNA-SCD1 (1070.0 ± 6.75 μM), in comparison with T98G/pcDNA3.1 (649.8 ± 8.40 μM), and an ∼2.4-fold increase in U87/pcDNA-SCD1 (1325 ± 14.58 μM), in comparison with U87/pcDNA3.1 (553.2 ± 14.91 μM) (**Figures 3D,E**). We also knocked down the expression of SCD1 by siRNA in TMZ-resistant T98G-R and U87-R cells (**Figures 3F,G**). We found that upon knocking-down of SCD1, T98G-R cells showed greater sensitivity to TMZ, presenting an ∼4-fold decrease in IC_50_ (T98G-R/si-Ctl: 1829.0 ± 19.33 μM, T98G-R/si-SCD1: 409.8 ± 6.36 μM) (**Figure 3H**). Similar results were observed in U87-R cells (**Figure 3I**). Given that programmed cell death might have key roles in chemotherapeutic responses (Leduc and Quoix, 2017), we wanted to access whether SCD1 might have an influence on the cell apoptosis and necrosis. Unexpectedly, compared with the untreated group, no obvious changes of apoptotic and necrotic cells could be seen upon SCD1 upregulation or downregulation (**Supplementary Figures S1A,B**), suggesting the effect of SCD1 on the chemosensitivity is independent on the cell apoptosis and necrosis. In addition, as DNA damage response has been proved to provoke the induction of chemoresistance in human cancer cells (Gilbert and Hemann, 2010), we evaluated the association between SCD1 expression and DNA damage biomarkers, such as positive modulator MGMT and negative modulator γ-H2AX (Wang et al., 2013). The data from Gliovis webserver (Bowman et al., 2017) showed a positive correlation between the expression of SCD1 and MGMT in GBM patients (**Supplementary Figure S2A**). Overexpression of SCD1 significantly upregulated MGMT level in T98G and U87 cells, and knocked down of SCD1 by siRNA downregulated MGMT in T98G-R and U87-R cells (**Supplementary Figure S2B**). But inconsistent changing trends could be seen in the γ-H2AX level (**Supplementary Figures S2C,D**). Confocal analysis showed more reduced γ-H2AX foci in T98G-R and U87-R cells, compared with the parent cells, further supporting the chemoresistance phenotype. When SCD1 over-expressed or knocked down, the corresponding γ-H2AX fluorescence signal in cells became much stronger or weaker, respectively (**Supplementary Figures S2E–H**). In brief, these data collectively demonstrate that SCD1 expression modulates TMZ resistance via the DNA damage response pathway: increased SCD1 may confer resistance to TMZ in GBM parental cells, while inhibition of SCD1 could re-sensitize TMZ-resistant GBM cells to TMZ.

**FIGURE 4:**
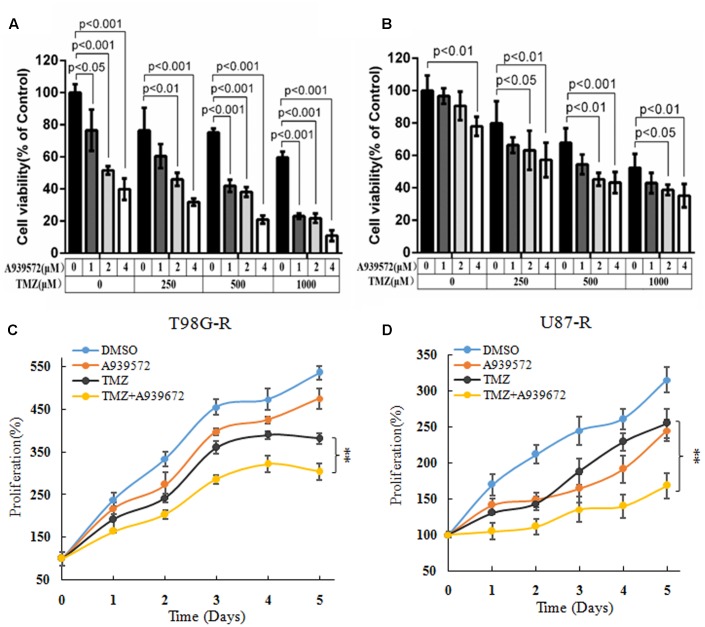
Inhibition of SCD1 by A939572 sensitizes TMZ-resistant GBM cells to TMZ. **(A,B)** T98G-R and U87-R cell viability was examined after treatment with A939572, TMZ or A939572 plus TMZ with the indicated concentrations for 72 h. **(C,D)** *In vitro* cell proliferation was analyzed after treating cells with single agents or combination treatment for 5 days. Experiments were repeated three times with similar results. ^∗∗^*p* < 0.01.

A939572 is a small specific inhibitor against SCD1 enzymatic activity (Bednarski et al., 2016). To further demonstrate the chemosensitizing effect of SCD1 inhibition, T98G-R and U87-R cells were treated with a combination of A939572 and TMZ. Compared with treatment with only TMZ, combined treatment showed a significant growth inhibition in a dose-dependent manner in T98G-R and U87-R cells. Moreover, TMZ combined with A939572 was much more effective in inhibiting cell proliferation compared with either agent alone (**Figures 4A,B**). The combination group yielded a reduction in cell viability within 5 days of treatment compared to the blank control group (**Figures 4C,D**). Similar change tendency of the MGMT transcriptional level could be clearly seen in the combination group (**Supplementary Figure S3**). All together, these data suggest that the inhibition of SCD1 may be an effective method to increase the TMZ sensitivity of GBM cells.

### SCD1 Promotes the Akt/GSK3β/β-Catenin Signaling Pathway

**FIGURE 5:**
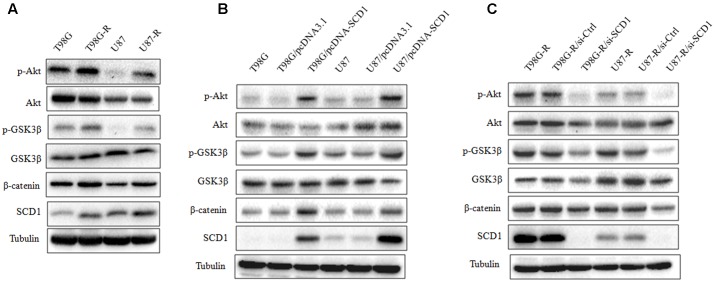
Effect of SCD1 on the Akt signaling pathway. **(A)** Western blot for the Akt signaling related proteins, Akt, GSK3β, and β-catenin in T98G, T98G-R, U87, and U87-R cells. **(B)** After T98G and U87 cells were transfected with pcDNA3.1 or pcDNA-SCD1 for 48 h, the total protein was extracted and analyzed by western blot with the indicated antibodies. **(C)** After T87G-R and U87-R cells were transfected with si-Ctrl or si-SCD1 for 48 h, the total protein was extracted and analyzed by western blot with the indicated antibodies. α-Tubulin was used as the internal control. All western blots are representative of results from at least three independent experiments.

**FIGURE 6:**
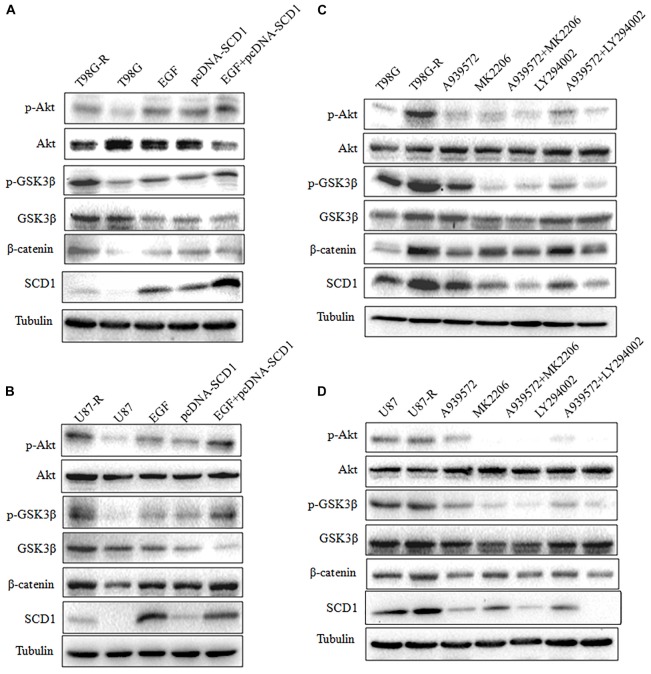
Modulation of Akt activation level has a feedback effect on the SCD1 level. Western blot for phospho-Akt (Ser-473), Akt, phospho-GSK3β (Ser-9), GSK3β, β-catenin, and SCD1 in T98G and U87 cells treated with pcDNA3.1, pcDNA-SCD1, EGF or pcDNA-SCD1 plus EGF **(A,B)**, or T98G-R and U87-R treated with DMSO, A939572, MK2206, A939572 plus MK2206, LY294002 and A939572 plus LY294002 **(C,D)**. All western blots are representative of results from at least three independent experiments. And α-Tubulin was used as the internal control.

Recent studies have demonstrated that SCD1 modulates several cancer-associated signaling pathways, such as Akt pathways (Nashed et al., 2012). Hence, we explored whether SCD1 mediates TMZ resistance through an Akt-dependent mechanism. Indeed, we found that phosphorylated Akt, as well as two downstream factors (phosphorylated GSK3β and β-catenin), were significantly upregulated in TMZ-resistant cells (**Figure 5A**). We then assessed the effect of SCD1 on Akt, GSK3β and β-catenin. We observed that overexpression of SCD1 significantly upregulated phosphorylation of Akt at Ser-473, phosphorylation of GSK-3β at Ser-9 and β-catenin in T98G and U87 cells (**Figure 5B**). In contrast, levels of phosphorylated Akt, phosphorylated GSK3β and β-catenin were reduced in si-SCD1-transfected T98G-R and U87-R cells (**Figure 5C**). To further clarify the SCD1-mediated Akt signaling axis in TMZ-resistant glioma cells, we used the Akt activator, EGF, to activate Akt signaling (Zhou et al., 2012), and the Akt direct inhibitor (MK2206) and indirect inhibitor (LY294002) to inactivate Akt signaling (Xu et al., 2017). We observed that activating Akt by EGF promoted SCD1-mediated upregulation of p-GSK3β and β-catenin in T98G and U87 cell lines (**Figures 6A,B**). Inactivation of Akt using inhibitors LY294002 and MK2206 could elevate the SCD1 inhibition-mediated downregulation of p-GSK3β and β-catenin in T98G-R and U87-R cell lines (**Figures 6C,D**). Meanwhile, both Akt activation and inhibition could have feedback effects on the SCD1 expression level (**Figures 6A–D**). These results suggest that SCD1 promotes Akt/GSK3β/β-catenin signaling in TMZ-resistant glioma cells.

### Inhibition of Akt/GSK3β/β-Catenin Signaling Attenuates SCD1-Mediated TMZ Resistance

**FIGURE 7:**
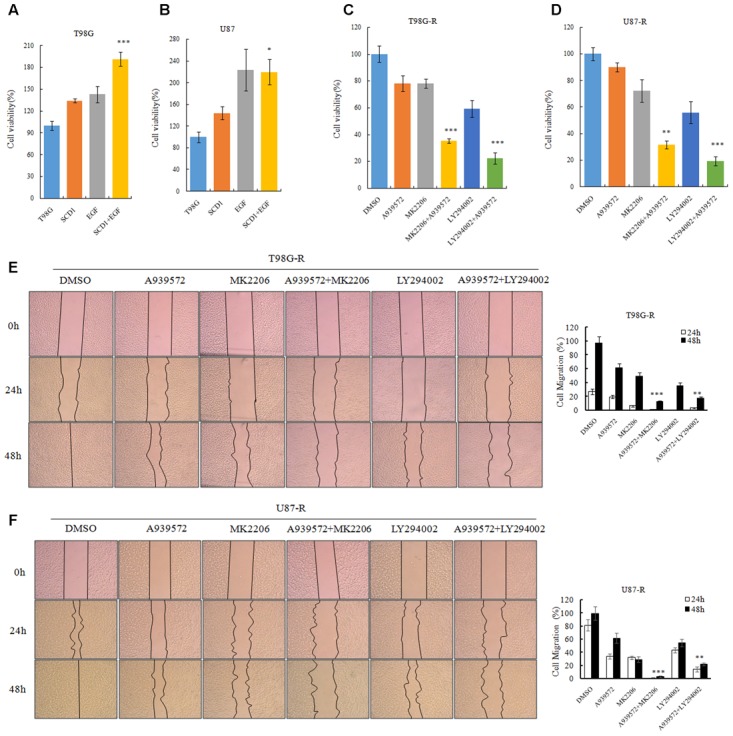
Akt/GSK3β/β-catenin signaling promotes SCD1-induced TMZ resistance. **(A,B)** T98G and U87 cells treated with pcDNA-SCD1, EGF or pcDNA-SCD1 plus EGF subjected to MTS assays. **(C,D)** Cell viability assays were performed after treating T98G-R and U87-R cells with anSCD1 inhibitor (A939572), Akt inhibitors (MK2206 and LY294002) or a combination of A939572 and Akt inhibitors. **(E,F)** Wound healing assay images captured after treating T98G-R and U87-R cells with A939572, MK2206, LY294002 or a combination of A939572 and Akt inhibitors for 0, 24, and 48 h. Experiments were repeated three times with similar results. ^∗^*p* < 0.05, ^∗∗^*p* < 0.01, ^∗∗∗^*p* < 0.001.

To evaluate whether the TMZ resistance-promoting effect of SCD1 was through an Akt-dependent mechanism, we first examined cell viability of GBM cells under pcDNA-SCD1 transfection and EGF treatment. Compared to the non-treatment group, T98G and U87 cells showed a greater viability when treated with exogenous SCD1 and EGF (**Figures 7A,B**). Next, we used an SCD1 inhibitor and Akt inhibitors to examine the cell viability and migration capacity of TMZ-resistant GBM cells. As shown in **Figures 7C,D**, the T98G-R and -R cells posed a modest decrease in cell viability when treated with an SCD1 inhibitor (A939572) and a 20∼40% decrease with Akt inhibitors treatment. These inhibitory effects were remarkably increased by combinational treatment. We performed a wound healing migration assay and observed that T98G-R cells treated with an SCD1 inhibitor and Akt inhibitors had a significantly lower rate of wound closure compared with the control group and either of the individual treatment groups (**Figure 7E**). Similar results were obtained in U87-R cell lines (**Figure 7F**). The increased inhibitory effect on cell growth and migration further supports the notion that anti-tumor activity by SCD1 inhibition is a consequence of the inhibition of Akt signaling.

## Discussion

The mechanisms contributing to TMZ resistance in GBM are still not clearly defined. Cancer cells, distinct from non-neoplastic cells, require large amounts of ATP and macromolecules to sustain rapid proliferation and division. Enhanced glycolysis, increased glutamine metabolism and elevated lipogenesis have been recognized as characteristic changes of cancer cells. TMZ-resistant cells escape the chemotoxicity of TMZ by a series of defense mechanisms that demand a large supply of metabolic substrates. The increased dependency of TMZ-resistant cells on cancer metabolism reminds us that blocking metabolic pathways might offer a promising strategy to preferentially kill intractable cancer cells.

**FIGURE 8:**
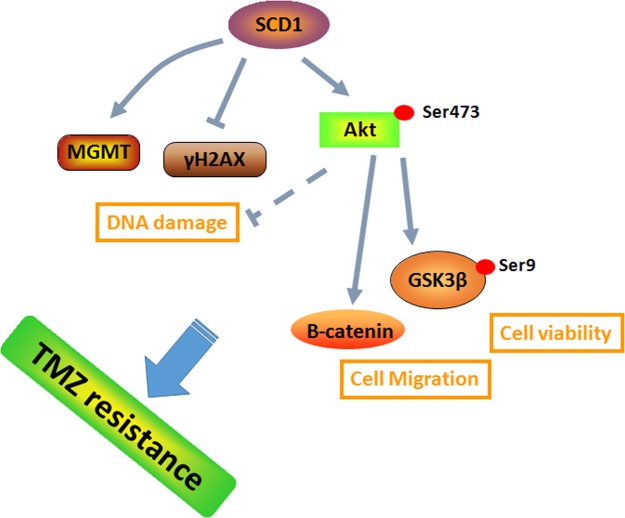
A schematic for TMZ resistance induced by SCD1 mediated the activation of Akt/GSK3β/β-catenin signaling axis. The upregulated SCD1 has pro-growth and pro-migration effects via activating the Akt/GSK3β/β-catenin signaling pathway, resulting in the TMZ resistance phenotype in GBM cells.

Previous studies suggested that metabolic reprogramming in cancer cells contributes to chemoresistance (Guerra et al., 2017; Qian et al., 2017). However, it is still unknown whether the switch of energetic dependency contributes to TMZ resistance. Therefore, we conducted a targeted metabolic PCR array to examine whether TMZ-resistant cells have altered metabolism. The results showed that some metabolic enzymes involved in aerobic glycolysis, glutaminolysis, and lipogenesis are altered. Our data identified SCD1 as the key factor in TMZ-induced metabolic alteration, indicating that SCD1 is related to TMZ resistance in GBM cells. We demonstrated that overexpression of SCD1 enhanced the resistance of the parental GBM cells to TMZ, while downregulation of SCD1 by siRNA or inhibitor (A939572) treatment led to increased sensitivity to TMZ in TMZ-resistant cell lines (seen the schematic in **Figure 8**). These data imply that metabolic reprogramming of GBM cells in adaptation to TMZ therapeutic stress might be a fundamental mechanism of TMZ resistance. Furthermore, TMZ-induced expression of SCD1 could serve as a novel mechanism for acquired resistance to TMZ.

The pivotal participation of SCD1 in tumor formation and cancer progression has been actively investigated in recent years. However, little is known regarding the involvement of SCD1 in GBM. Gopal et al. (1963) analyzed the fatty acid distribution in several intracranial tumors and showed that the levels of unsaturated fatty acids are considerably higher in gliomas than in normal brain, which might point to aberrant SCD1 activity in this disease. In addition, sterol response element binding protein-1 (SREBP-1), a transcription factor regulating multiple enzymes involved in fatty acid synthesis, was also highly activated in GBM patient tissues (Guo et al., 2009), which could lead to a high transcriptional activity of SCD1 in GBM. In our study, we firstly demonstrated that SCD1 could enhance the resistance to TMZ *in vitro*. Thus, a better understanding of the potential roles of SCD1 in GBM biology, especially the characteristics of glioma patients, is of great significance for the study of SCD1-targeted therapy. Additionally, further studies on metabolomics are essential to demonstrate whether the function of SCD1 in TMZ resistance is indeed a consequence of lipogenesis.

The Akt signaling pathway was demonstrated to be frequently hyperactivated in human cancers (Xu et al., 2016). Not only is the Akt signaling pathway responsible for a diverse set of biological processes, but it is also a master regulator of cancer metabolism (Priolo et al., 2014). Akt is a powerful inducer of lipogenesis in cancer cells, mainly through activating SREBP-1 (Guo et al., 2014). Additionally, it was observed that ablation of SCD1 expression decreases Akt phosphorylation and activity in cancer cells (Tan et al., 2014), which further supports the concept that SCD1 is required for Akt activation. SCD1 also controls cancer cell proliferation through the modulation of other downstream targets of Akt, such as GSK3β. Inhibition of SCD1 in cancer cells leads to dephosphorylation and activation of GSK3β, which halts cell proliferation by inducing β-catenin and cyclin D1 ubiquitinylation and degradation (Scaglia and Igal, 2008). This study is consistent with previous findings that overexpressed or silenced SCD1 promotes or inhibits Akt/GSK3β/β-catenin signaling, respectively. When treated with a combination of exogenous SCD1 and Akt activator, we found that the Akt/GSK3β/β-catenin signaling pathway was further activated. Meanwhile, the collaborative inhibitory effect to Akt/GSK3β/β-catenin signaling was observed by the combined inhibition of SCD1 and Akt. Functionally, SCD1 inhibitor could inhibit cell growth and migration of TMZ-resistant cells, while the inhibitory effects could be further observed when treated with a combination of an SCD1 inhibitor and Akt inhibitors. These results indicate that the pro-growth and pro-migration effects of SCD1 in TMZ-resistant cells are dependent on the Akt/GSK3β/β-catenin signaling pathway.

## Conclusion

Our study highlights the important roles of SCD1 in TMZ resistance and indicates that SCD1 inhibition could be an effective strategy for overcoming TMZ resistance in GBM chemotherapy. This study suggests that SCD1 may represent a therapeutic target for overcoming TMZ resistance in GBM patients.

## Author Contributions

SD and YY performed the experiment. ZX, SZ, and LQ wrote this article. LH, XL, LS, and ZG designed the study and contributed in manuscript preparation.

## Conflict of Interest Statement

The authors declare that the research was conducted in the absence of any commercial or financial relationships that could be construed as a potential conflict of interest.

## Supplementary Material

**FIGURE S1 |** No obvious changes of cell apoptosis and necrosis are modulated by SCD1. Flow cytometry was used to evaluate the effect of SCD1 on the cell apoptosis and necrosis in parental T98G and U87 cells **(A)**, and the TMZ resistant cells T98G-R and U87-R **(B)**.

**FIGURE S2 |** SCD1 inhibits the DNA damage repair in GBM cells. **(A)** The association between SCD1 and MGMT was analyzed from the Gliovis database. **(B)** After transfected with SCD1 siRNA or overexpression plasmid, qPCR was performed to detect the MGMT transcriptional level. **(C,D)** Western blot for the γ-H2AX in GBM cells with transfected with SCD1 siRNA or overexpression plasmid. **(E,F)** Fluorescence confocal microscopy was used to evaluate the γ-H2AX foci in GBM cells with transfected with SCD1 siRNA or overexpression plasmid. Representative images are shown. Scale bar = 10 μm. **(G,H)** Quantification of γ-H2AX fluorescent puncta is shown. Bars represent the means ± SD of triplicate samples. ^∗^*p* < 0.05, ^∗∗^*p* < 0.01.

**FIGURE S3 |** Inhibition of SCD1 attenuate the MGMT mRNA expression. After treatment with A939572, TMZ or A939572 plus TMZ with the indicated concentrations for 72 h, qPCR was used to analyze the change of MGMT transcriptional level. The experiments were performed three times, and one representative result was shown. ^∗∗^Indicated the significant difference.

**TABLE S1 |** The detailed primer information used for the metabolism-focused PCR array.
