# Corrigendum: SCD1 Confers Temozolomide Resistance to Human Glioma Cells *Via the* Akt/GSK3β/β-Catenin Signaling Axis

**DOI:** 10.3389/fphar.2019.01358

**Published:** 2019-11-15

**Authors:** Shuang Dai, Yuanliang Yan, Zhijie Xu, Shuangshuang Zeng, Long Qian, Lei Huo, Xuejun Li, Lunquan Sun, Zhicheng Gong

**Affiliations:** ^1^Department of Pharmacy, Xiangya Hospital, Central South University, Changsha, China; ^2^School of Pharmaceutical Sciences, Central South University, Changsha, China; ^3^National Clinical Research Center for Geriatric Disorders, Xiangya Hospital, Central South University, Changsha, China; ^4^Department of Pathology, Xiangya Hospital, Central South University, Changsha, China; ^5^Department of Neurosurgery, Xiangya Hospital, Central South University, Changsha, China; ^6^Center for Molecular Medicine, Key Laboratory for Molecular Radiation Oncology of Hunan Province, Xiangya Hospital, Central South University, Changsha, China

**Keywords:** glioblastomas, temozolomide, resistance, SCD1, Akt signaling

In the original article, there was a mistake in **Figure 6D** as published. The final submission for the SCD1 bands in **Figure 6D** was inadvertently truncated too short. The corrected **Figure 6D** appears below.

**Figure 6:**
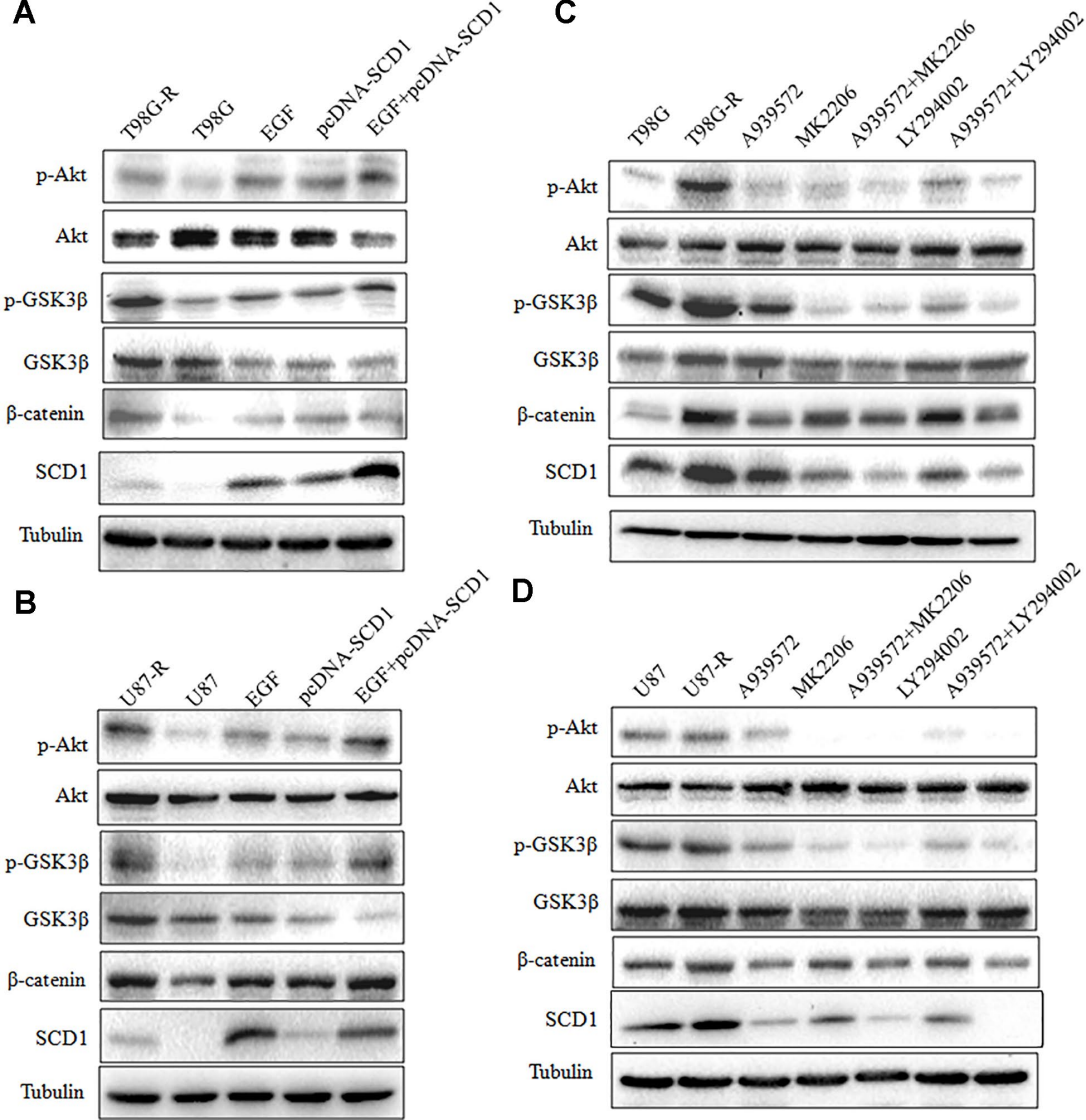
Modulation of Akt activation level has a feedback effect on the SCD1 level. Western blot for phospho-Akt (Ser-473), Akt, phospho-GSK3b (Ser-9),GSK3β, β-catenin, and SCD1 in T98G and U87 cells treated with pcDNA3.1, pcDNA-SCD1, EGF or pcDNA-SCD1 plus EGF **(A, B)**, or T98G-R and U87-R treated with DMSO, A939572, MK2206, A939572 plus MK2206, LY294002 and A939572 plus LY294002 **(C, D)**. All western blots are representative of results from at least three independent experiments. And α-Tubulin was used as the internal control.

The authors apologize for this error and state that this does not change the scientific conclusions of the article in any way. The original article has been updated.

