# Supplementary figures and images for: SCD1 Confers Temozolomide Resistance to Human Glioma Cells via the Akt/GSK3β/β-Catenin Signaling Axis
Source: Front Pharmacol. 2018 Jan 4;8:960. doi: 10.3389/fphar.2017.00960 (PMC5758607; doi:10.3389/fphar.2017.00960)

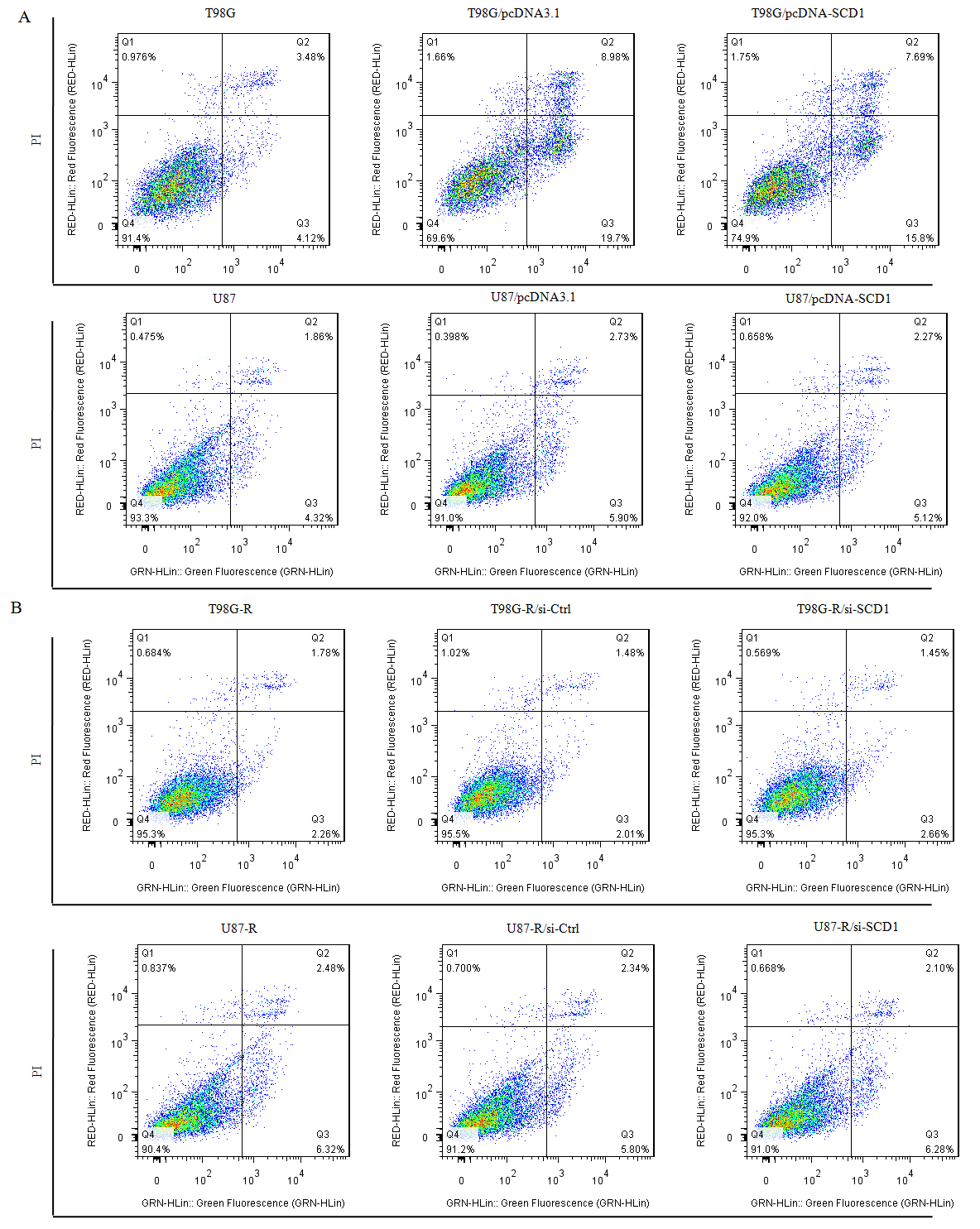

Supplement: Supplementary file 1 [file Image_1.TIF]

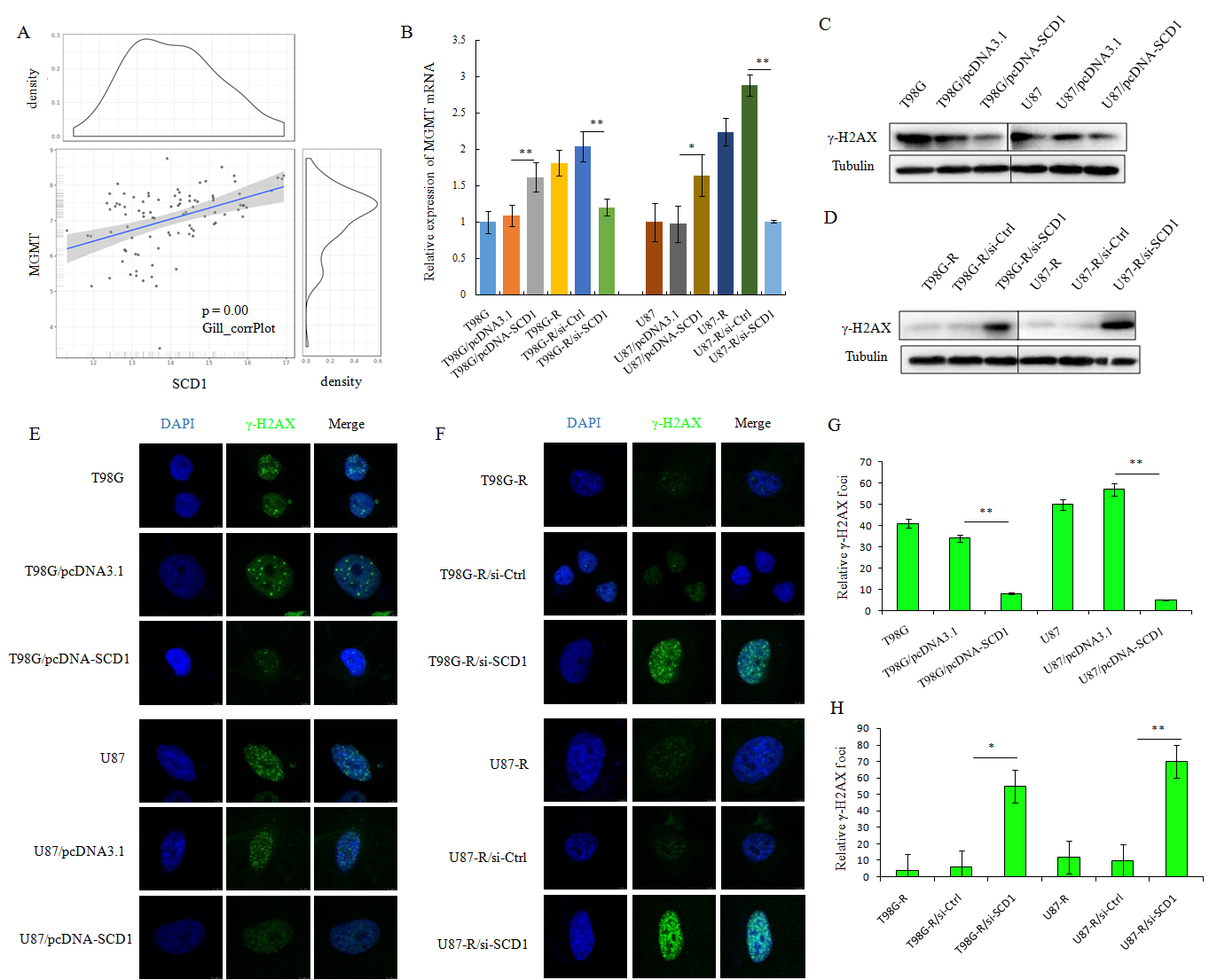

Supplement: Supplementary file 2 [file Image_2.TIF]

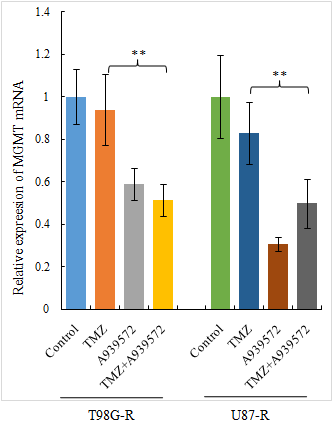

Supplement: Supplementary file 3 [file Image_3.TIF]
