# Supplementary material for: SCD1 Confers Temozolomide Resistance to Human Glioma Cells via the Akt/GSK3β/β-Catenin Signaling Axis
Source: Front Pharmacol. 2018 Jan 4;8:960. doi: 10.3389/fphar.2017.00960 (PMC5758607; doi:10.3389/fphar.2017.00960)
Supplement: Supplementary file 4 [file Table_1.DOC]

**Supplementary Table 1. The detailed primer information used for the metabolism-focused PCR array.**

| Accession No. | Gene Symbol | Forward Primer | Reverse Primer |
| --- | --- | --- | --- |
| [NM_001145145.1](http://www.ncbi.nlm.nih.gov/entrez/query.fcgi?cmd=Search&db=Nucleotide&term=NM_001145145) | SLC1A5 | TCATGTGGTACGCCCCTGT | GCGGGCAAAGAGTAAACCCA |
| [NM_198838.1](http://www.ncbi.nlm.nih.gov/entrez/query.fcgi?cmd=Search&db=Nucleotide&term=NM_198838) | ACC | CATGCGGTCTATCCGTAGGTG | GTGTGACCATGACAACGAATCT |
| [NM_002197.2](http://www.ncbi.nlm.nih.gov/entrez/query.fcgi?cmd=Search&db=Nucleotide&term=NM_002197) | ACO1 | CGCAGCACAAGAACATAGAAGT | CATTGCAGCAAAGTCAACCAC |
| [NM_001098.2](http://www.ncbi.nlm.nih.gov/entrez/query.fcgi?cmd=Search&db=Nucleotide&term=NM_001098) | ACO2 | TTGAGCCCAACGAGTACATCC | GTCCATACACAATCTTCTCCGAG |
| [NM_000045.3](http://www.ncbi.nlm.nih.gov/entrez/query.fcgi?cmd=Search&db=Nucleotide&term=NM_000045) | ARG1 | GTGGAAACTTGCATGGACAAC | AATCCTGGCACATCGGGAATC |
| [NM_001172.3](http://www.ncbi.nlm.nih.gov/entrez/query.fcgi?cmd=Search&db=Nucleotide&term=NM_001172) | ARG2 | TGACATCAACACACCCCTTACC | GTCCACGTCTCTCAGACCAAT |
| [NM_001024944.1](http://www.ncbi.nlm.nih.gov/entrez/query.fcgi?cmd=Search&db=Nucleotide&term=NM_001024944) | ASAL | CAGTGGACCCCATCATGGAGA | GGCTTTGCTGCCTTGAACATC |
| [NM_000050.4](http://www.ncbi.nlm.nih.gov/entrez/query.fcgi?cmd=Search&db=Nucleotide&term=NM_000050) | ASS1 | CTTGGGGCCAAAAAGGTGTTC | GAGGTAGCGGTCCTCATACAG |
| [NM_001216.2](http://www.ncbi.nlm.nih.gov/entrez/query.fcgi?cmd=Search&db=Nucleotide&term=NM_001216) | CA9 | CTGTCTCGCTTGGAAGAAATCG | GCAGGAGTGCAGATATGTCCAG |
| [NM_000387.5](http://www.ncbi.nlm.nih.gov/entrez/query.fcgi?cmd=Search&db=Nucleotide&term=NM_000387) | CACT | GACCAGCCAAAACCCATCAG | AGAGGGTGACCGACGAACA |
| [NM_001122633.2](http://www.ncbi.nlm.nih.gov/entrez/query.fcgi?cmd=Search&db=Nucleotide&term=NM_001122633) | CPSI | AATGAGGTGGGCTTAAAGCAAG | AGTTCCACTCCACAGTTCAGA |
| [NM_004077.2](http://www.ncbi.nlm.nih.gov/entrez/query.fcgi?cmd=Search&db=Nucleotide&term=NM_004077) | CS | AACTGCTACCCAAGGCTAAGG | CTTTTGAGAGCCAAGATACCTGT |
| [NM_001931.4](http://www.ncbi.nlm.nih.gov/entrez/query.fcgi?cmd=Search&db=Nucleotide&term=NM_001931) | DLAT | CGGAACTCCACGAGTGACC | CCCCGCCATACCCTGTAGT |
| [NM_000108.4](http://www.ncbi.nlm.nih.gov/entrez/query.fcgi?cmd=Search&db=Nucleotide&term=NM_000108) | DLD | CACTGCTACGAAAGCTGATGG | TAACTTCTGAACCCGTGGCTA |
| [NM_004104.4](http://www.ncbi.nlm.nih.gov/entrez/query.fcgi?cmd=Search&db=Nucleotide&term=NM_004104) | FASN | AAGGACCTGTCTAGGTTTGATGC | TGGCTTCATAGGTGACTTCCA |
| [NM_000143.3](http://www.ncbi.nlm.nih.gov/entrez/query.fcgi?cmd=Search&db=Nucleotide&term=NM_000143) | FH | CGGTCAGGTCTGGGAGAATTG | ACACTGAGTAGGGTTCACCTT |
| [NM_000151.3](http://www.ncbi.nlm.nih.gov/entrez/query.fcgi?cmd=Search&db=Nucleotide&term=NM_000151) | G6PC | ACTGGCTCAACCTCGTCTTTA | CGGAAGTGTTGCTGTAGTAGTCA |
| [NM_001081686.1](http://www.ncbi.nlm.nih.gov/entrez/query.fcgi?cmd=Search&db=Nucleotide&term=NM_001081686) | G6PC2 | CCCAAATCACTCAAGTCCATGC | GGTTACCATGACATACCAGACAC |
| [NM_138387.3](http://www.ncbi.nlm.nih.gov/entrez/query.fcgi?cmd=Search&db=Nucleotide&term=NM_138387) | G6PC3 | GTAAGGGTGATGCCTAGCCTG | TGGTGAGGGAAATGTGCTAAGAT |
| [NM_001256310.1](http://www.ncbi.nlm.nih.gov/entrez/query.fcgi?cmd=Search&db=Nucleotide&term=NM_001256310) | GLS1 | TCTACAGGATTGCGAACGTCT | CTTTGTCTAGCATGACACCATCT |
| [NM_013267.3](http://www.ncbi.nlm.nih.gov/entrez/query.fcgi?cmd=Search&db=Nucleotide&term=NM_013267) | GLS2 | TCTCTTCCGAAAGTGTGTGAGC | CCGTGAACTCCTCAAAATCAGG |
| [NM_005271.4](http://www.ncbi.nlm.nih.gov/sites/entrez?db=nuccore&cmd=search&term=NM_005271) | GLUD | CTGGCTTGGCATACACAATG | GCTGTTCTCAGGTCCAATCC |
| [NM_002065.6](https://www.ncbi.nlm.nih.gov/nuccore/742068537) | GLUL | TAAGGACCCTAACAAGCTGGT | CCGTTTACAGGTGTGCCTCAA |
| NC_000001 | GLUT | AGCAGGAGACCAAACGACG | GCGCGACTCACCTTGCT |
| [NM_002079.2](https://www.ncbi.nlm.nih.gov/nuccore/197304792) | GOT1 | ATTTCTTAGCGCGTTGGTACA | ACACAGCATTGTGATTCTCCC |
| [NM_002080.3](http://www.ncbi.nlm.nih.gov/entrez/query.fcgi?cmd=Search&db=Nucleotide&term=NM_002080) | GOT2 | AAGAGGGACACCAATAGCAAAAA | GCAGAACGTAAGGCTTTCCAT |
| [NM_005309.2](http://www.ncbi.nlm.nih.gov/sites/entrez?db=nuccore&cmd=search&term=NM_005309) | GPT1 | CAGCGGGAAGGCACCTA | GTGAACTTGGCATGGAACCT |
| [NM_133443.3](http://www.ncbi.nlm.nih.gov/entrez/query.fcgi?cmd=Search&db=Nucleotide&term=NM_133443) | GPT2 | GACCCCGACAACATCTACCTG | TCATCACACCTGTCCGTGACT |
| [NM_002088.4](http://www.ncbi.nlm.nih.gov/entrez/query.fcgi?cmd=Search&db=Nucleotide&term=NM_002088) | GRIK5 | GATCAACGGGATCATCGAGGT | GTGTCCGTGGTCTCGTACTG |
| [NM_145657.2](http://www.ncbi.nlm.nih.gov/entrez/query.fcgi?cmd=Search&db=Nucleotide&term=NM_145657) | GSX1 | GCGCTCTACCAGACCTCCTA | CGGGACAGGTACATATTAGAAGC |
| [NM_133267.2](http://www.ncbi.nlm.nih.gov/entrez/query.fcgi?cmd=Search&db=Nucleotide&term=NM_133267) | GSX2 | ATGTCGCGCTCCTTCTATGTC | CAAGCGGGATGAAGAAATCCG |
| [NM_021957.3](http://www.ncbi.nlm.nih.gov/entrez/query.fcgi?cmd=Search&db=Nucleotide&term=NM_021957) | GSY2 | GTGGAACAGTGTGAACCTGTAA | AGGACTTCCTTCTATCAGCCAT |
| [NM_001161587.1](http://www.ncbi.nlm.nih.gov/entrez/query.fcgi?cmd=Search&db=Nucleotide&term=NM_001161587) | GYS1 | CAGCGCGGACCAACAATTTC | TCCTCCCGAACTTTTCCTTCA |
| [NM_033498.2](http://www.ncbi.nlm.nih.gov/entrez/query.fcgi?cmd=Search&db=Nucleotide&term=NM_033498) | HK1 | GCTCTCCGATGAAACTCTCATAG | GGACCTTACGAATGTTGGCAA |
| [NM_000189.4](http://www.ncbi.nlm.nih.gov/entrez/query.fcgi?cmd=Search&db=Nucleotide&term=NM_000189) | HK2 | TGCCACCAGACTAAACTAGACG | CCCGTGCCCACAATGAGAC |
| [NM_002115.2](http://www.ncbi.nlm.nih.gov/entrez/query.fcgi?cmd=Search&db=Nucleotide&term=NM_002115) | HK3 | GGACAGGAGCACCCTCATTTC | CCTCCGAATGGCATCTCTCAG |
| [NM_033508.1](http://www.ncbi.nlm.nih.gov/entrez/query.fcgi?cmd=Search&db=Nucleotide&term=NM_033508) | HK4 | GCAGAAGGGAACAATGTCGTG | CGTAGTAGCAGGAGATCATCGT |
| [NM_001166059.1](http://www.ncbi.nlm.nih.gov/entrez/query.fcgi?cmd=Search&db=Nucleotide&term=NM_001166059) | HMGCL | GTGTCTCCTAAGTGGGTTCCC | TGGGTAGTTGATGCCAGGAAA |
| [NM_001130996.1](http://www.ncbi.nlm.nih.gov/entrez/query.fcgi?cmd=Search&db=Nucleotide&term=NM_001130996) | HMGCR | TGATTGACCTTTCCAGAGCAAG | CTAAAATTGCCATTCCACGAGC |
| [NM_001098272.2](http://www.ncbi.nlm.nih.gov/entrez/query.fcgi?cmd=Search&db=Nucleotide&term=NM_001098272) | HMGCS | CTCTTGGGATGGACGGTATGC | GCTCCAACTCCACCTGTAGG |
| [NM_001166107.1](http://www.ncbi.nlm.nih.gov/entrez/query.fcgi?cmd=Search&db=Nucleotide&term=NM_001166107) | HMGCS2 | GCCCAATATGTGGACCAAACT | GAAGCCCATACGGGTCTGG |
| [NM_005896.3](http://www.ncbi.nlm.nih.gov/entrez/query.fcgi?cmd=Search&db=Nucleotide&term=NM_005896) | IDH1 | AGAAGCATAATGTTGGCGTCA | CGTATGGTGCCATTTGGTGATT |
| [NM_002168.3](http://www.ncbi.nlm.nih.gov/entrez/query.fcgi?cmd=Search&db=Nucleotide&term=NM_002168) | IDH2 | CGCCACTATGCCGACAAAAG | ACTGCCAGATAATACGGGTCA |
| [NM_001165415.1](http://www.ncbi.nlm.nih.gov/entrez/query.fcgi?cmd=Search&db=Nucleotide&term=NM_001165415) | LDHA | ATGGCAACTCTAAAGGATCAGC | CCAACCCCAACAACTGTAATCT |
| [NM_002300.7](http://www.ncbi.nlm.nih.gov/entrez/query.fcgi?cmd=Search&db=Nucleotide&term=NM_002300) | LDHB | TCTGTGACCGCCAATTCTAAGA | GCACCAGATTGAGCCGACTC |
| [NM_017448.3](http://www.ncbi.nlm.nih.gov/entrez/query.fcgi?cmd=Search&db=Nucleotide&term=NM_017448) | LDHC | AGAACATGGTGATTCTAGTGTGC | ACAGTCCAATAGCCCAAGAGG |
| [NM_003051.3](http://www.ncbi.nlm.nih.gov/entrez/query.fcgi?cmd=Search&db=Nucleotide&term=NM_003051) | MCT1 | AGGTCCAGTTGGATACACCCC | GCATAAGAGAAGCCGATGGAAAT |
| [NM_001201548.1](http://www.ncbi.nlm.nih.gov/entrez/query.fcgi?cmd=Search&db=Nucleotide&term=NM_001201548) | MCT4 | CTTTAGCCACCACATTTCCACT | AGGCAGTATCAATGCCAGGTAA |
| [NM_001199112.1](http://www.ncbi.nlm.nih.gov/entrez/query.fcgi?cmd=Search&db=Nucleotide&term=NM_001199112) | MDH1 | ACCATGCCAAGGTGAAATTGC | ACAGTCGTGACAAATTCTCCC |
| [NM_005918.3](http://www.ncbi.nlm.nih.gov/entrez/query.fcgi?cmd=Search&db=Nucleotide&term=NM_005918) | MDH2 | GCCATGATCTGCGTCATTGC | CCGAAGATTTTGTTGGGGTTGT |
| [NM_002395.5](http://www.ncbi.nlm.nih.gov/entrez/query.fcgi?cmd=Search&db=Nucleotide&term=NM_002395) | ME1 | GAGTGCTGACATCTGACATTGA | TTGGCTTCCGAAACACCAAAC |
| [NM_002396.4](http://www.ncbi.nlm.nih.gov/entrez/query.fcgi?cmd=Search&db=Nucleotide&term=NM_002396) | ME2 | GTGCAGGAATACGGCCTGATA | AATGGGTCTTTTAAGAGTGCGAT |
| [NM_003047.4](http://www.ncbi.nlm.nih.gov/entrez/query.fcgi?cmd=Search&db=Nucleotide&term=NM_003047) | NHE1 | CACCTCCCGATTTACCTCCCA | GGCTGACAAGTAGGCCATGT |
| [NM_001003941.2](http://www.ncbi.nlm.nih.gov/entrez/query.fcgi?cmd=Search&db=Nucleotide&term=NM_001003941) | OGDH | GGCTTCCCAGACTGTTAAGAC | GCAGAATAGCACCGAATCTGTTG |
| [NM_000531.5](http://www.ncbi.nlm.nih.gov/entrez/query.fcgi?cmd=Search&db=Nucleotide&term=NM_000531) | OTC | CGGCCCGTGTATTGTCTAGC | TAGCCAGGGTGTCCAAATCTG |
| [NM_001040716.1](http://www.ncbi.nlm.nih.gov/entrez/query.fcgi?cmd=Search&db=Nucleotide&term=NM_001040716) | PC | GCTGGAGGAGAATTACACCCG | GGATGTTCCCATACTGGTCCC |
| [NM_000284.3](http://www.ncbi.nlm.nih.gov/entrez/query.fcgi?cmd=Search&db=Nucleotide&term=NM_000284) | PDHA1 | ATGGAATGGGAACGTCTGTTG | CCTCTCGGACGCACAGGATA |
| [NM_005390.4](http://www.ncbi.nlm.nih.gov/entrez/query.fcgi?cmd=Search&db=Nucleotide&term=NM_005390) | PDHA2 | ATTCGCGGTTTCTGTCACCTG | GTGATCCGAGGGGTTTATGCC |
| [NM_001173468.1](http://www.ncbi.nlm.nih.gov/entrez/query.fcgi?cmd=Search&db=Nucleotide&term=NM_001173468) | PDHB | AAGAGGCGCTTTCACTGGAC | ACTAACCTTGTATGCCCCATCA |
| NM_001278549.1 | PDK1 | CTGTGATACGGATCAGAAACCG | TCCACCAAACAATAAAGAGTGCT |
| [NM_001323016.1](https://www.ncbi.nlm.nih.gov/nuccore/1019366839) | PFKFB3 | TTGGCGTCCCCACAAAAGT | AGTTGTAGGAGCTGTACTGCTT |
| [NM_001166686.1](http://www.ncbi.nlm.nih.gov/entrez/query.fcgi?cmd=Search&db=Nucleotide&term=NM_001166686) | PFKM | AGCGTTTCGATGATGCTTCAG | GGAGTCGTCCTTCTCGTTCC |
| [NM_000290.3](https://www.ncbi.nlm.nih.gov/nuccore/259490363) | PGAM2 | AGAAGCACCCCTACTACAACTC | TCTGGGGAACAATCTCCTCGT |
| [NM_000291.3](https://www.ncbi.nlm.nih.gov/nuccore/183603937) | PGK1 | TGGACGTTAAAGGGAAGCGG | GCTCATAAGGACTACCGACTTGG |
| [NM_006623.3](http://www.ncbi.nlm.nih.gov/entrez/query.fcgi?cmd=Search&db=Nucleotide&term=NM_006623) | PHGDH | CTGCGGAAAGTGCTCATCAGT | TGGCAGAGCGAACAATAAGGC |
| NM_182471.3 | PKM | AAGGGTGTGAACCTTCCTGG | GCTCGACCCCAAACTTCAGA |
| [NM_005609.3](http://www.ncbi.nlm.nih.gov/entrez/query.fcgi?cmd=Search&db=Nucleotide&term=NM_005609) | PYGM | CCATGCCCTACGATACGCC | TAGCCACCGACATTGAAGTCC |
| [NM_005063.4](http://www.ncbi.nlm.nih.gov/entrez/query.fcgi?cmd=Search&db=Nucleotide&term=NM_005063) | SCD1 | TCTAGCTCCTATACCACCACCA | TCGTCTCCAACTTATCTCCTCC |
| [NM_004168.3](http://www.ncbi.nlm.nih.gov/entrez/query.fcgi?cmd=Search&db=Nucleotide&term=NM_004168) | SDHA | TGGCATTTCTACGACACCGTG | GCCTGCTCCGTCATGTAGTG |
| [NM_003000.2](http://www.ncbi.nlm.nih.gov/entrez/query.fcgi?cmd=Search&db=Nucleotide&term=NM_003000) | SDHB | ACCTTCCGAAGATCATGCAGA | GTGCAAGCTAGAGTGTTGCCT |
| [NM_003001.3](http://www.ncbi.nlm.nih.gov/entrez/query.fcgi?cmd=Search&db=Nucleotide&term=NM_003001) | SDHC | TAGGTTCAAACCGTCCTCTGT | GAGAGACCCCTGCACTCAAAG |
| [NM_003002.3](http://www.ncbi.nlm.nih.gov/entrez/query.fcgi?cmd=Search&db=Nucleotide&term=NM_003002) | SDHD | ATTTCTTCAGGACCGACCTATCC | CAGCCTTGGAGCCAGAATG |
| [NM_020346.2](http://www.ncbi.nlm.nih.gov/entrez/query.fcgi?cmd=Search&db=Nucleotide&term=NM_020346) | SLC17A6 | TGGACATGGTCAACAACAGCA | GGAACCGTGGATCATCCCC |
| [NM_020309.3](http://www.ncbi.nlm.nih.gov/entrez/query.fcgi?cmd=Search&db=Nucleotide&term=NM_020309) | SLC17A7 | CTGGGGCTACATTGTCACTCA | GCAAAGCCGAAAACTCTGTTG |
| [NM_001145288.1](http://www.ncbi.nlm.nih.gov/entrez/query.fcgi?cmd=Search&db=Nucleotide&term=NM_001145288) | SLC17A8 | CCTCCCCAAGCGTTACATCAT | GCTGTCTGAATTTCCGGTTTTCC |
| [NM_004170.5](http://www.ncbi.nlm.nih.gov/entrez/query.fcgi?cmd=Search&db=Nucleotide&term=NM_004170) | SLC1A1 | TTCTAATGCGGATGCTGAAACT | CGCGCAGACCAATTTTTCC |
| [NM_004171.3](http://www.ncbi.nlm.nih.gov/entrez/query.fcgi?cmd=Search&db=Nucleotide&term=NM_004171) | SLC1A2 | TGTCCACGACCATCATTGCTG | TTCTTGAGCTTGGGATTGCCT |
| [NM_001166696.2](http://www.ncbi.nlm.nih.gov/entrez/query.fcgi?cmd=Search&db=Nucleotide&term=NM_001166696) | SLC1A3 | AGCAGGGAGTCCGTAAACG | AGCATTCCGAAACAGGTAACTTT |
| [NM_005071.2](../../../../C:%5C学术association%5C期刊投稿资料%5C戴爽SCD1%20论文%5Cassay_template.xls) | SLC1A6 | TGCGCCCATATCAGCTCAC | CAATGAGAGGTAACACCAGCAT |
| [NM_006671.5](http://www.ncbi.nlm.nih.gov/entrez/query.fcgi?cmd=Search&db=Nucleotide&term=NM_006671) | SLC1A7 | TTCAAACAGTACCGCACCAAG | CTGGACCCCGTAGATGAGGA |
| [NM_138817.3](http://www.ncbi.nlm.nih.gov/entrez/query.fcgi?cmd=Search&db=Nucleotide&term=NM_138817) | SLC7A13 | TGCTTTACACTTATAGCAGGGGA | AGTCACCAGAGGTAACGCAGT |
| [NM_003486.6](http://www.ncbi.nlm.nih.gov/entrez/query.fcgi?cmd=Search&db=Nucleotide&term=NM_003486) | SLC7A5 | GGAAGGGTGATGTGTCCAATC | TAATGCCAGCACAATGTTCCC |
